# The formation of electronically excited species in the human multiple myeloma cell suspension

**DOI:** 10.1038/srep08882

**Published:** 2015-03-06

**Authors:** Marek Rác, Michaela Sedlářová, Pavel Pospíšil

**Affiliations:** 1 Department of Biophysics, Centre of the Region Haná for Biotechnological and Agricultural Research, Faculty of Science, Palacký University, Šlechtitelů 11, 783 71 Olomouc, Czech Republic; 2 Department of Botany, Faculty of Science, Palacký University, Šlechtitelů 11, 783 71 Olomouc, Czech Republic

## Abstract

In this study, evidence is provided on the formation of electronically excited species in human multiple myeloma cells U266 in the growth medium exposed to hydrogen peroxide (H_2_O_2_). Two-dimensional imaging of ultra-weak photon emission using highly sensitive charge coupled device camera revealed that the addition of H_2_O_2_ to cell suspension caused the formation of triplet excited carbonyls ^3^(R = O)*. The kinetics of ^3^(R = O)* formation in the real time, as measured by one-dimensional ultra-weak photon emission using low-noise photomultiplier, showed immediate enhancement followed by a slow decay. In parallel to the formation of ^3^(R = O)*, the formation of singlet oxygen (^1^O_2_) in U266 cells caused by the addition of H_2_O_2_ was visualized by the imaging of ^1^O_2_ using the green fluorescence of singlet oxygen sensor green detected by confocal laser scanning microscopy. Additionally, the formation of ^1^O_2_ after the addition of H_2_O_2_ to cell suspension was detected by electron paramagnetic resonance spin-trapping spectroscopy using 2,2,6,6-tetramethyl-4-piperidone. Presented results indicate that the addition of H_2_O_2_ to cell suspension results in the formation of ^3^(R = O)* and ^1^O_2_ in U266 cell suspension. The contribution of the cell-free medium to the formation of electronically excited species was discussed.

Reactive oxygen species (ROS) are produced as a byproduct of either metabolic processes such as cellular respiration in mitochondria and photosynthesis in chloroplast^1^,^2^,^3^ or oxidative burst in phagocytic cells known to play role in the defense against infection^4^. The one-electron reduction of molecular oxygen produces superoxide anion radical (O_2_^•−^) known to dismutate to hydrogen peroxide (H_2_O_2_) and subsequently to hydroxyl radical (HO^•^). When ROS are not sufficiently eliminated by non-enzymatic (low molecular weight scavengers) and enzymatic (the superoxide dismutase and peroxidase families of enzymes) defense systems, ROS oxidize biomolecules comprising lipid, protein and nucleic acid^5^,^6^,^7^,^8^. The oxidation of lipid and protein by abstraction of weakly bonded hydrogen atom by HO^•^ results in the formation of lipid and protein alkyl radicals (R^•^), respectively. Subsequently, lipid and protein peroxyl radicals (ROO^•^) are formed by the interaction of R^•^ with molecular oxygen. Peroxyl radicals abstract hydrogen from lipid and protein, while lipid and protein hydroperoxides (ROOH) are formed. Under reducing conditions (reduced transition metals such as Fe^2+^, Mn^2+^, Zn^2+^ or Cu^+^ bound in metalloproteins, transport proteins, and storage proteins), ROOH is reduced to lipid and protein alkoxyl radicals (RO^•^), whereas under oxidizing conditions (oxidized transition metals, cytochrome c, peroxynitrite, chloroperoxide, and hypochlorous acid), ROOH is oxidized back to lipid and protein ROO^•^^9^,^10^.

The oxidation of biomolecules by ROS was proposed to be associated with the formation of electronically excited species such as triplet excited carbonyl ^3^(R = O)* and singlet oxygen (^1^O_2_)^11^,^12^,^13^,^14^. Based on chemical system experiments, ^3^(R = O)* was proposed to be produced by the decomposition of cyclic (1,2-dioxetane, ROOR) and linear (tetroxide, ROOOOR) high-energy intermediates. 1,2-dioxetane is formed by the cycloaddition of ^1^O_2_ to lipid and protein, by the cyclization of lipid and protein ROO^•^^15^, and by the enzymatic reactions^16^,^17^,^18^. Tetroxide is formed by the recombination of two lipid and protein ROO^•^ via Russell mechanism^15^,^19^. Once ^3^(R = O)* is formed, the excitation energy of ^3^(R = O)* can be transferred either to molecular oxygen resulting in the formation of ^1^O_2_^20^ or to chromophore (C) forming singlet excited chromophore (^1^C*)^14^. Beside the reactions related to the transfer of excitation energy, ^3^(R = O)* is known to undergo a variety of other reactions comprising of isomerization, cleavage, hydrogen abstraction, and cycloaddition^21^,^22^. In addition to ^3^(R = O)* pathway, the direct decomposition of ROOOOR to ^1^O_2_ by Russell mechanisms was evidenced in the chemical system^23^,^24^. More recently, several lines of evidence were provided on the formation of ^1^O_2_ by the decomposition of lipid and protein ROOH by metal ions, cytochrome c, peroxynitrite, chloroperoxide, and hypochlorous acid^25^,^26^,^27^,^28^. As the yield of ^3^(R = O)* formation during the decomposition of ROOOOR was found to be 10^3^–10^4^ lower compared to the yield of ^1^O_2_ formation, it was proposed that the Russell mechanism leads predominantly to the formation of ^1^O_2_, whereas the formation of ^3^(R = O)* is rather negligible^29^.

The excitation energy of electronically excited species formed under oxidative processes is emitted as ultra-weak photon emission^14^,^30^,^31^,^32^. The excitation energy of ^3^(R = O)* is emitted as near UVA and blue-green photons in the spectral range of 350–550 nm^14^,^19^. The photon emission of ^1^C* is in the green-red region of the spectrum (550–750 nm)^14^, whereas dimol and monomol photon emission of ^1^O_2_ is in the red (634, 703 nm) and infra-red (1270 nm) region of the spectrum^33^,^34^, respectively. Recent development in one-dimensional detection of ultra-weak photon emission using low-noise photomultiplier tubes (PMT) and two-dimensional detection of ultra-weak photon emission using highly sensitive charge coupled device (CCD) cameras allows temporal and spatial visualization of the formation of electronically excited species.

In spite of the fact that several lines of evidence were provided on the formation of ^3^(R = O)* and ^1^O_2_ in chemical system, a limited number of studies have focused on the production of electronically excited species in cells. In this study, experimental evidence on the formation of ^3^(R = O)* and ^1^O_2_ in human multiple myeloma cell line U266 suspended in the growth medium containing free amino acids, fetal bovine serum and traces of transition metal ions is provided. The detection of the electronically excited species was performed using ultra-weak photon emission, confocal laser scanning microscopy and electron paramagnetic (EPR) spin-trapping spectroscopy. It is demonstrated here that both ^3^(R = O)* and ^1^O_2_ are formed in the cell suspension exposed to oxidative environment.

## Results

### The effect of various H_2_O_2_ concentrations on the formation of the electronically excited species and cell viability

**Figure 1:**
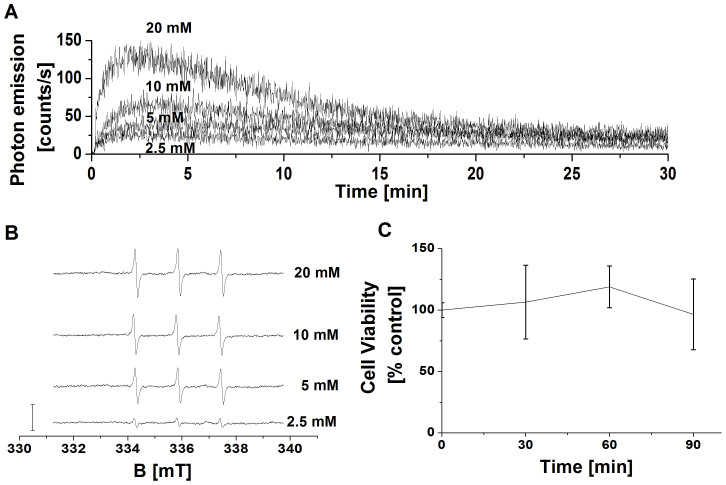
The effect of various concentrations of H_2_O_2_ on the formation of electronically excited species. In (A), detection of one-dimensional ultra-weak photon emission. One-dimensional ultra-weak photon emission from cell suspension was measured by low-noise PMT after the addition of H_2_O_2_ at concentrations indicated in the figure. In (B), detection of TEMPONE EPR signal by EPR spin-trapping spectroscopy. TEMPONE EPR spectra were detected from cell suspension treated with H_2_O_2_ for 30 min at the concentrations indicated in the figure. 50 mM TEMPD was added to cell suspension prior to the measurement. The bar represents 3000 relative units. In (C), determination of cell viability by automated cell counter. The cell suspension was treated with 5 mM H_2_O_2_ for the time period indicated in the figure. The data are presented as the mean and standard deviation of 3 measurements (mean ± SD, n = 3).

To select the most suitable concentration of H_2_O_2_ for the formation of ^3^(R = O)* and ^1^O_2_, the effect of various H_2_O_2_ concentrations on the formation of ^3^(R = O)* and ^1^O_2_ was tested using one-dimensional ultra-weak photon emission and EPR spin-trapping spectroscopy, respectively. Figure 1A shows the one-dimensional ultra-weak photon emission measured from cell suspension after the addition of H_2_O_2_ at different concentrations. The addition of H_2_O_2_ at different concentrations to the cell suspension resulted in the immediate significant increase of one-dimensional ultra-weak photon emission as compared to the untreated cell suspension. In further, the formation of TEMPONE EPR signal in cell suspension was measured after the addition of H_2_O_2_ at various concentrations. Figure 1B shows that TEMPONE EPR signal was enhanced nonlinearly with the increasing concentration of H_2_O_2_. These observations reveal that the addition of 5 mM H_2_O_2_ to the cell suspension results in sufficient response allowing the detection of electronically excited species. To test whether 5 mM H_2_O_2_ affects the viability of cells, the number of live cells was determined after the addition of 5 mM H_2_O_2_. Figure 1C shows that the cell viability remained unaffected by the addition of 5 mM H_2_O_2_ to the cell suspension. Based on the results from one-dimensional ultra-weak photon emission, EPR spin-trapping spectroscopy, and cell viability, the concentration of 5 mM H_2_O_2_ was chosen for further experiments.

### The detection of triplet excited carbonyls by two-dimensional ultra-weak photon emission

**Figure 2:**
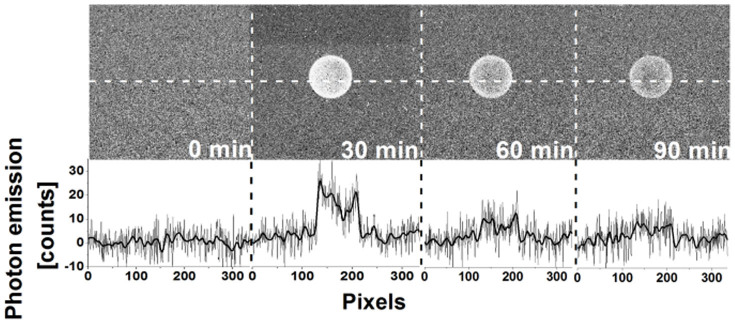
Triplet excited carbonyl detection by two-dimensional imaging of ultra-weak photon emission. Two-dimensional ultra-weak photon emission was measured from the cell suspension by a highly sensitive CCD camera. Prior to the measurements, the cell suspension was kept in the dark for 10 min. After dark period, two-dimensional ultra-weak photon emission was measured from untreated cell suspension. Consequently, a set of three images of two-dimensional ultra-weak photon emission was measured after the addition of H_2_O_2_ to cell suspension for time period indicated in the figure. The two-dimensional ultra-weak photon emission was measured with the integration time of 30 min. The bottom panel shows the spatial profile of photon emission in the middle strip of the image. Y axis denotes the number of counts accumulated after 30 min, whereas X axis denotes the pixel of the image.

To visualize the formation of ^3^(R = O)* in cell suspension treated with H_2_O_2_, the two-dimensional ultra-weak photon emission was examined using a highly sensitive CCD camera. Figure 2 shows the two-dimensional ultra-weak photon emission measured from cell suspension after the addition of H_2_O_2_. The treatment of cell suspension with H_2_O_2_ resulted in the pronounced increase in two-dimensional ultra-weak photon emission compared to control. To quantify the differences in two-dimensional ultra-weak photon emission from cell suspension treated with H_2_O_2_, the spatial profile of photon emission in the middle strip of the image was used. The number of counts of two-dimensional ultra-weak photon emission from cell suspension measured in the absence of H_2_O_2_ was under the detection limit of CCD camera. The intensity of two-dimensional ultra-weak photon emission was highest during the first 30 min. These results indicate that the addition of H_2_O_2_ to cell suspension causes the formation of ^3^(R = O)*.

### Detection of triplet excited carbonyls by one-dimensional ultra-weak photon emission

**Figure 3:**
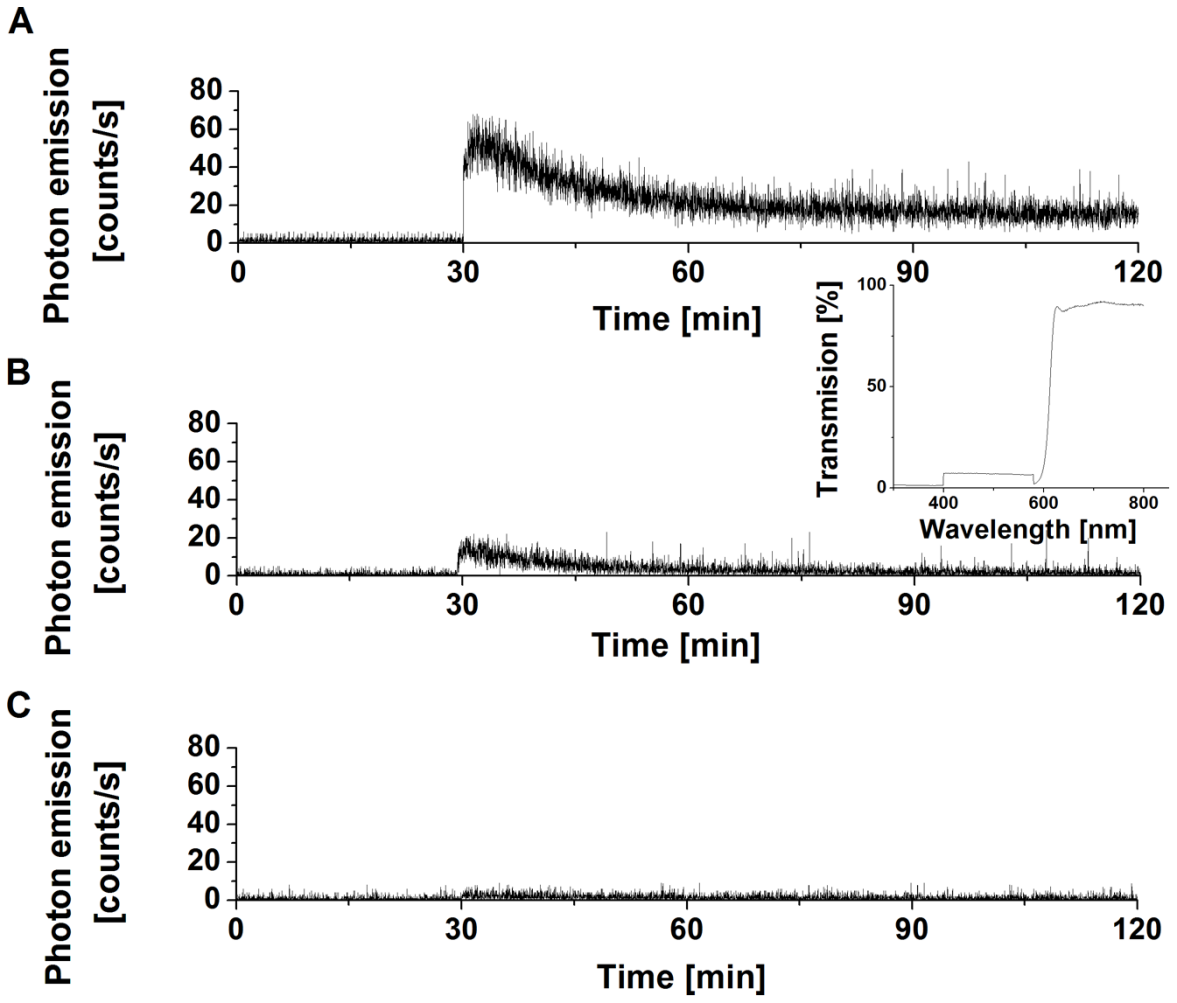
Triplet excited carbonyl detection by one-dimensional ultra-weak photon emission. One-dimensional ultra-weak photon emission from cell suspension was measured by low-noise PMT. In (A), spontaneous one-dimensional ultra-weak photon emission was measured for 30 min. Consequently, 5 mM H_2_O_2_ was added to the cell suspension. In (B), the one-dimensional ultra-weak photon emission from cell suspension treated with 5 mM H_2_O_2_ was measured in the presence of the long-pass edge filter (600 nm). Insert shows transmission spectrum of long-pass edge filter. In (C), the effect of 10 mM histidine on one-dimensional ultra-weak photon emission from cell suspension is shown. Other experimental conditions were as in Fig. 2B.

To study the kinetics of ultra-weak photon emission, one-dimensional ultra-weak photon emission was measured using low-noise PMT. Spontaneous one-dimensional ultra-weak photon emission from cell suspension measured in the absence of H_2_O_2_ shows a steady-state level, whereas the addition of H_2_O_2_ after 30 min induced pronounced enhancement of one-dimensional ultra-weak photon emission followed by slow decay (Fig. 3A). To confirm that one-dimensional ultra-weak photon emission is in the region of the spectrum assigned to ^3^(R = O)*, the one-dimensional ultra-weak photon emission was measured using long-pass edge filter (600 nm). Figure 3B shows that one-dimensional ultra-weak photon emission was significantly suppressed in the presence of the long-pass edge filter. To determine the participation of ^1^O_2_ in one-dimensional ultra-weak photon emission originated from red region of the spectrum, the effect of histidine, ^1^O_2_ scavenger, on one-dimensional ultra-weak photon was measured. Figure 3C demonstrates that the one-dimensional ultra-weak photon emission in the red region was almost fully suppressed in the presence of histidine. These results prove that most of the one-dimensional ultra-weak photon emission originates from the blue-green region of the spectrum known to be associated with the photon emission of ^3^(R = O)*. Moreover, the measurement of one-dimensional ultra-weak photon emission reveals that the formation of ^3^(R = O)* starts immediately after the addition of H_2_O_2_ to the cell suspension.

### Formation of singlet oxygen detected by confocal laser scanning microscopy

**Figure 4:**
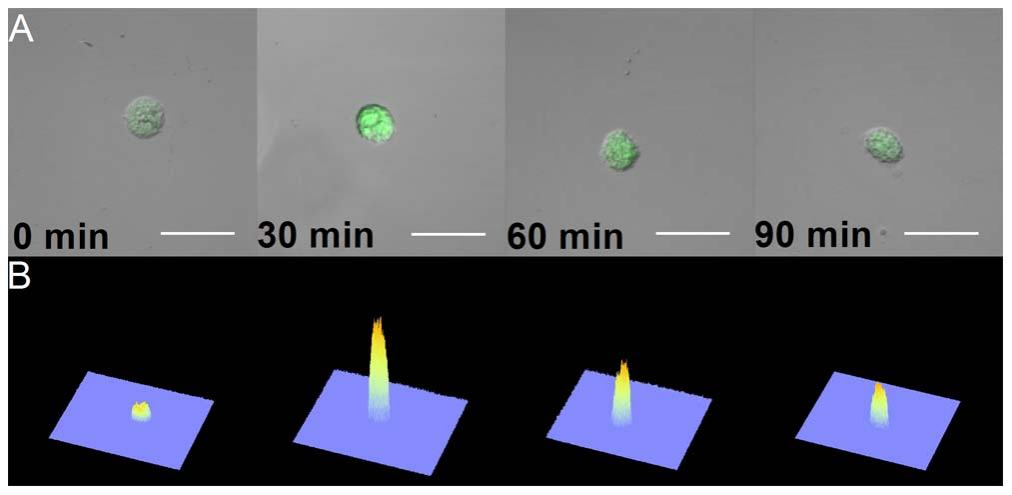
Singlet oxygen imaging by confocal laser scanning microscopy. The SOSG fluorescence within U266 cells treated with 5 mM H_2_O_2_ for the time period indicated in the figure was examined by a confocal laser scanning microscope. 50 μM SOSG was added to U266 cells 30 min prior to the data collection. In (A), individual representative cells of each time variant are shown in the images combining Nomarski DIC and SOSG fluorescence (λ_em_ = 505–525 nm) channels. In (B), the integral distribution of the SOSG fluorescence intensity is shown within the corresponding upper images. The bar represents 30 μm.

**Figure 5:**
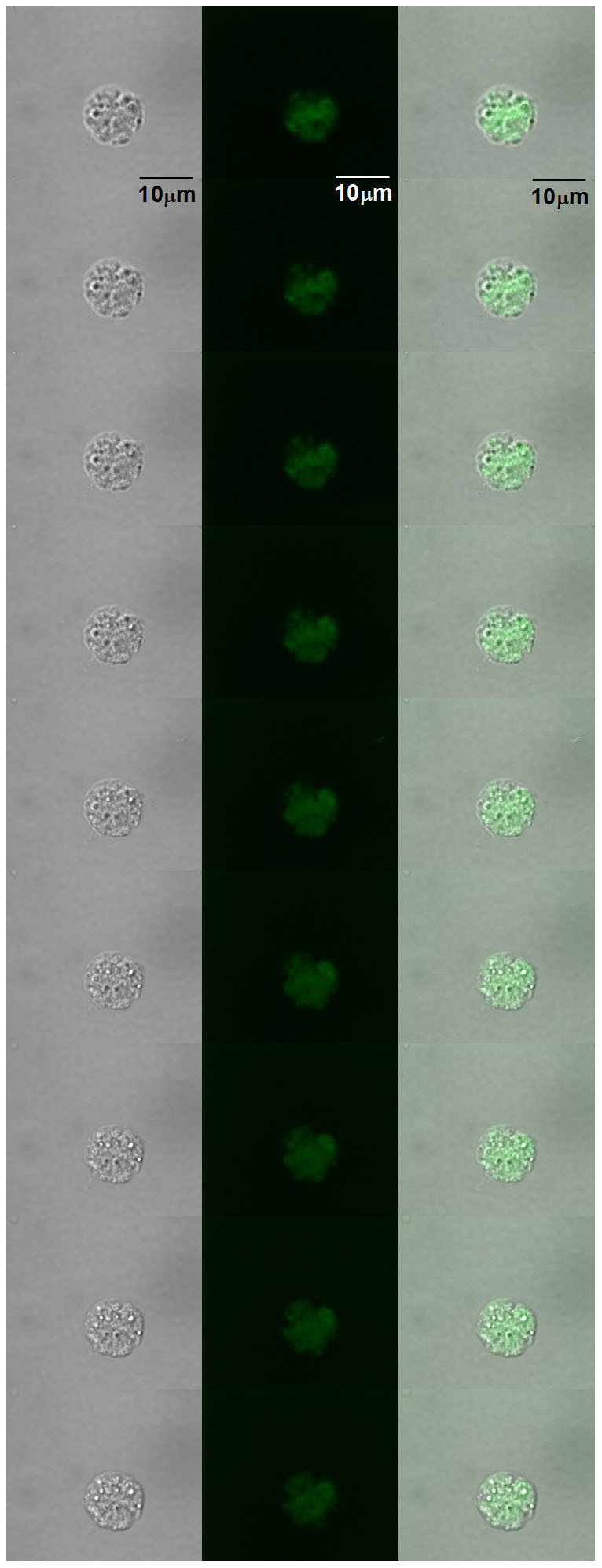
Singlet oxygen imaging in the multiple layers of the sample. U266 cells were treated with 5 mM H_2_O_2_ for 30 min. Three channels are presented: Nomarski DIC (left column), SOSG fluorescence (λ_em_ = 505–525 nm) (middle column) and the combination of Nomarski DIC and SOSG fluorescence (right column). The step in between different pictures is 0.5 μm. Other parameters are same as in Fig. 4.

To visualize the formation of ^1^O_2_ in U266 cells caused by the addition of H_2_O_2_, singlet oxygen sensor green (SOSG) fluorescence was detected by confocal laser scanning microscopy. In spite of its high selectivity to ^1^O_2_ without any undesirable interaction with O_2_^•−^ and HO^•^^35^, the SOSG has been reported to suffer from self-photosensitization^35^. To prevent the formation of ^1^O_2_ by SOSG itself, the dye was strictly protected from light exposition during the whole experiment. Figure 4A shows the combination of channels for SOSG fluorescence (in green) and Nomarski DIC (greyscale) of U266 cells treated with H_2_O_2_ for 0, 30, 60 and 90 min. Negligible SOSG fluorescence detected from untreated U266 cells was due to the weak SOSG fluorescence upon excitation by light at 480 nm prior to the reaction with ^1^O_2_. The formation of SOSG endoperoxide by the cycloaddition of ^1^O_2_ precludes the intramolecular electron transport thus resulting in the enhancement of the fluorescence. The intensity of SOSG fluorescence was pronouncedly higher in the H_2_O_2_-treated U266 cells compared to untreated U266 cells. Figure 4B represents the integral distribution of SOSG fluorescence intensity within the corresponding upper image. Although SOSG was originally devised for extracellular applications, several studies reported its penetration inside of cells^36^,^37^. To test whether SOSG penetrates into U266 cells, SOSG fluorescence was measured in the multiple layers of sample (Fig. 5). The presence of SOSG fluorescence in the multiple layers of sample reveals the formation of ^1^O_2_ inside of U266 cells after the addition of H_2_O_2_.

### Formation of singlet oxygen detected by EPR spin-trapping spectroscopy

**Figure 6:**
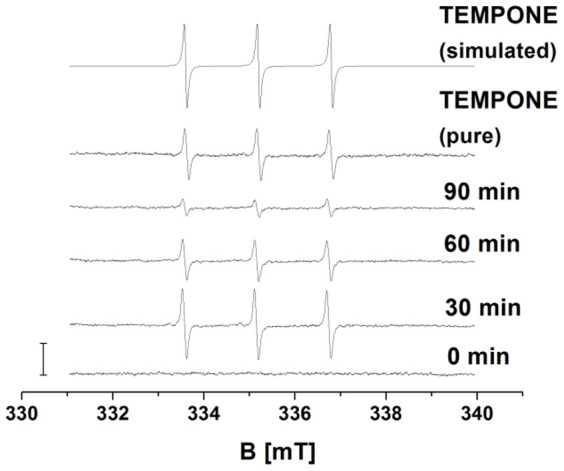
Detection of singlet oxygen by EPR spin-trapping spectroscopy. TEMPONE EPR spectra were detected from cell suspension treated with 5 mM H_2_O_2_ for the period indicated in the figure. 50 mM TEMPD was added to cell suspension 30 min prior to the measurement. Pure TEMPONE EPR signal was detected using 20 nM TEMPONE. The simulation of TEMPONE EPR spectra was done using hyperfine splitting constant *a*^N^ = 16 G. The bar represents 3000 relative units.

To study the kinetics of ^1^O_2_ formation in the cell suspension, EPR spin-trapping spectroscopy was used. The spin-trapping was accomplished by utilizing the oxidation of hydrophilic diamagnetic 2,2,6,6-tetramethyl-4-piperidone (TMPD) by ^1^O_2_ known to yield paramagnetic 2, 2, 6, 6-tetramethyl-4-piperidone-1-oxyl (TEMPONE). The observation that no TEMPONE EPR signal was detected in untreated cell suspension indicates that no ^1^O_2_ was formed in the untreated cell suspension (Fig. 6). The addition of H_2_O_2_ to the cell suspension caused the formation of TEMPONE EPR signal. To confirm that EPR signal is attributed to TEMPONE, the EPR spectrum of pure TEMPONE was measured. The simulation of TEMPONE EPR spectra using one spectral component with the hyperfine coupling constant *a*^N^ = 16 G provided agreement with the hyperfine coupling constant described for TEMPONE^38^. These results indicate that the addition of H_2_O_2_ to cell suspension results in the formation of ^1^O_2_.

### Quantitative analysis of triplet excited carbonyls and singlet oxygen formation

**Figure 7:**
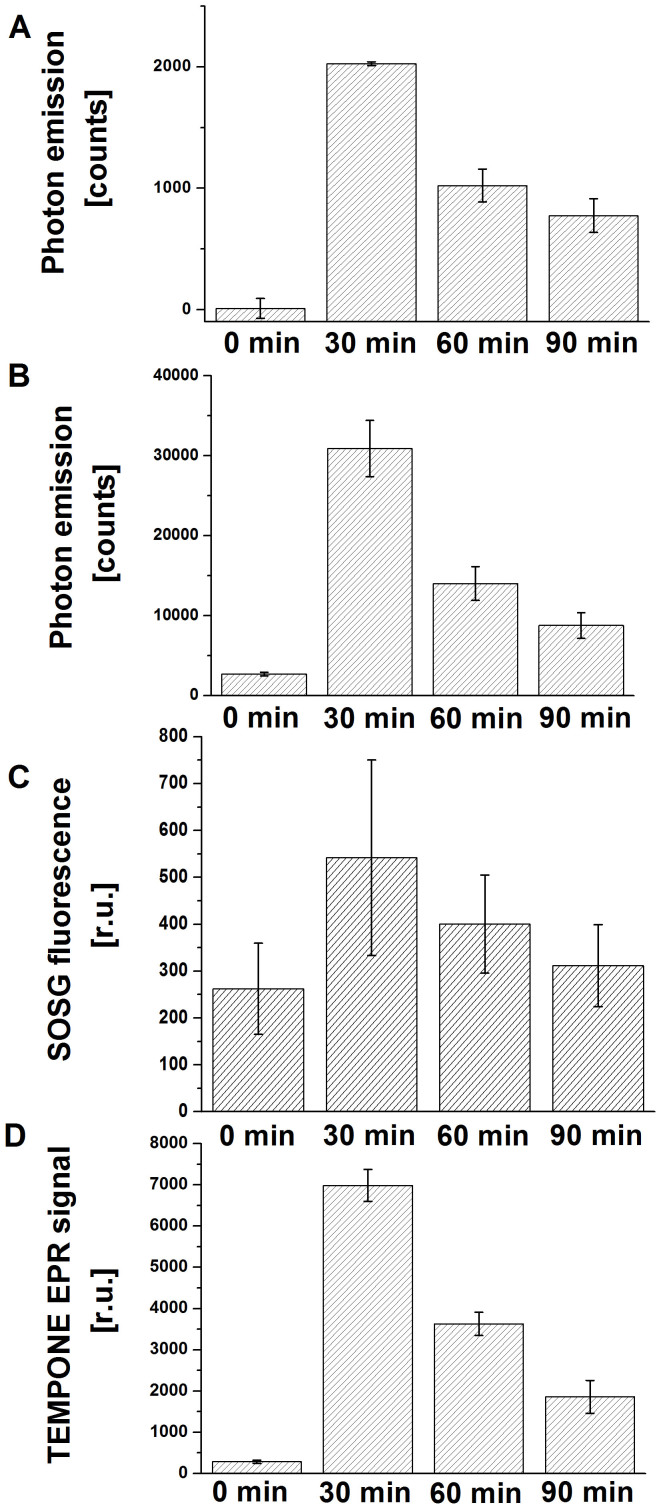
Quantitative analysis. Quantitative analysis of two-dimensional and one-dimensional ultra-weak photon emission, SOSG fluorescence intensity, and TEMPONE EPR signal. The spatial profile of ultra-weak photon emission in the middle trip of the image (A), the area below curve (B), the intensity of SOSG fluorescence (C) and the height of the middle peak of TEPONE EPR signal (D) measured from U266 cells was plotted as a function of the H_2_O_2_ treatment period indicated in the figure. The data are presented as the mean and standard deviation of at least 3 measurements (mean ± SD, n ≥ 3). The other experimental conditions were as in Figs. 1–4.

In order to quantify ^3^(R = O)* and ^1^O_2_, two-dimensional ultra-weak photon emission, one-dimensional ultra-weak photon emission, SOSG fluorescence and TEMPONE EPR signal were plotted as a function of H_2_O_2_ treatment period. To quantify two-dimensional images of ultra-weak photon emission from cell suspension treated with H_2_O_2_, the aera below the curve of spatial profile of photon emission at different strips of the image was counted. The intensity of the two-dimensional ultra-weak photon emission decreased by 50% and 62% after the addition of H_2_O_2_ for 60 and 90 min compared to 30 min (Fig. 7A). To evaluate one-dimensional ultra-weak photon emission from cell suspension treated with H_2_O_2_, the area below curve was counted over the 30 min of the treatment (Fig. 7B). Two-dimensional ultra-weak photon emission measured from the cell suspension decreased by 55% and 72% after the addition of H_2_O_2_ for 60 and 90 min compared to 30 min. To evaluate the SOSG fluorescence obtained using confocal laser scanning microscopy, the intensity of SOSG fluorescence within confocal images was analyzed by image analysis (Fig. 7C). Due to the high background of SOSG fluorescence, the intensity of SOSG fluorescence observed in untreated U266 cells was subtracted from the SOSG fluorescence observed in treated U266 cells prior to the evaluation. The SOSG fluorescence was decreased by 50% and 80% after the addition of H_2_O_2_ for 60 and 90 min compared to 30 min. The plotting of the height of the center peak of TEMPONE EPR spectrum against the time showed that TEMPONE EPR signal decreased by 50% and 75% after the addition of H_2_O_2_ for 60 and 90 min compared to 30 min (Fig. 7D). These results show that the formation of both ^3^(R = O)* and ^1^O_2_ decays rapidly shortly after the addition of H_2_O_2_ to cell suspension followed by slow decay over the whole time of experiment.

### The involvement of the medium in the formation of electronically excited species

**Figure 8:**
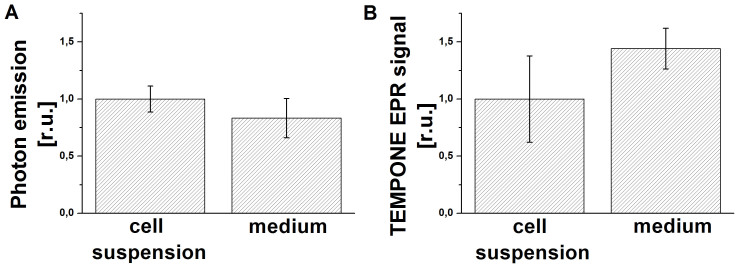
Formation of electronically excited species in the cell-free medium. The one-dimensional ultra-weak photon emission (A) and TEMPONE EPR signal (B) detected in the cell suspension and in the cell-free medium 30 min after the addition of 5 mM H_2_O_2_ is shown. In order to better compare the results, the sum of one-dimensional ultra-weak photon emission counts and the height of the middle peak of TEPONE EPR signal were normalized to the data from cell suspension.

In order to evaluate the effect of the medium on the formation of electronically excited species, the cell suspension was gently centrifuged and the supernatant was taken for further experiments. The addition of H_2_O_2_ to both cell suspension and cell-free medium resulted in significant increase in both the one-dimensional ultra-weak photon emission and TEMPONE EPR signal. The comparison of results revealed that the one-dimensional ultra-weak photon emission originated from the cell-free medium was lower by 17% compared to the cell suspension (Fig. 8A). On the other hand, the TEMPONE EPR signal detected from cell-free medium treated with H_2_O_2_ was higher by 44% compared to the TEMPONE EPR signal detected from cell suspension treated with H_2_O_2_ (Fig. 8B). These results suggest that the contribution of the cell-free medium to the formation of electronically excited species is not negligible.

## Discussion

Two-dimensional ultra-weak photon emission confirms the formation of ^3^(R = O)* upon the addition of H_2_O_2_ to cell suspension. Based on the results obtained by one-dimensional ultra-weak photon emission, it is concluded here that the formation of ^3^(R = O)* occurs immediately after the addition of H_2_O_2_ to cell suspension. Spectral analysis of the ultra-weak photon emission from different samples such as rat perfused lung, rat brain and liver homogenate, spinach mitochondria, cotyledons of etiolated seedlings of *Cicer arietinum* L., DNA, hemodialysis plasma cells, esophageal carcinoma cell line, porcine ex-vivo skin model induced by different treatment, showed that ultra-weak photon emission originates mostly from ^3^(R = O)*^14^,^43^,^44^,^45^,^46^,^47^,^48^. Several lines of evidence have been provided that ^3^(R = O)* is formed by decomposition of ROOR or ROOOOR^11^,^13^. The decomposition of ROOR involves the cleavage of oxygen-oxygen and carbon-carbon bonds by two different mechanisms. The concerted mechanism involves the simultaneous cleavage of oxygen-oxygen and carbon-carbon bonds. The diradical mechanism is a two-step reaction involving the cleavage of oxygen bound prior to the cleavage of carbon-carbon bond^15^. Tetroxide is formed by the recombination of two ROO^•^. It has been demonstrated that t-butyl hydroperoxide does not participate in the ROOOOR formation due to the fact that the presence of α-hydrogen is required in order to undergo the Russell pathway^24^,^49^. Based on this observation, it was established that solely the primary and secondary ROO^•^ are involved in the ROOOOR formation, whereas the tertiary ROO^•^ undergoes propagation of lipid peroxidation and protein oxidation. The conditions required for the Russell mechanism makes it rather unlikely, but not impossible, to occur in biological system.

Based on the data obtained from EPR spin-trapping spectroscopy itis concluded here that the formation of ^1^O_2_ occurs after the addition of H_2_O_2_ to cell suspension. The visualization of ^1^O_2_ formation in the multiple layers of sample confirmed that ^1^O_2_ was produced inside the U266 cells. These results clearly show that U266 cells are oxidized upon the exposure of cell suspension to H_2_O_2_ resulting in ^1^O_2_ formation. It has been previously demonstrated in the chemical systems that ^1^O_2_ is formed by the decomposition of organic hydroperoxides such as linoleic acid hydroperoxides, thymidine hydroperoxides, t-butyl hydroperoxides^9^,^50^,^51^,^52^. The decomposition of linoleic acid hydroperoxide formed by the photooxidation using methylene blue has been shown to result in the formation of ^1^O_2_ as confirmed by chemical trapping and monomol photon emission in near infra-red region^27^. The decomposition of ROOH results in the formation of ROO^•^ which leads to the formation of unstable ROOOOR known to decompose to ^1^O_2_, R = O, and ROH via the Russell mechanism^23^. The experimental evidence indicates that ^1^O_2_ is generated at a yield close to 10% by the Russell mechanism^52^. Furthermore, ^3^(R = O)* formed either through ROOR or ROOOOR pathway can transfer the excitation energy to molecular oxygen resulting in the formation of ^1^O_2_. Other possible pathways for ^1^O_2_ formation comprising the base-catalyzed reactions of H_2_O_2_, reaction of peroxynitrites with H_2_O_2_, reaction of H_2_O_2_ with hypochloride, and reactions involving enzymes (peroxidases and oxygenases) cannot be excluded.

The comparison of the formation of electronically excited species in the cell suspension and in the cell-free medium revealed that the addition of H_2_O_2_ to the cell suspension resulted in the higher formation of ^3^(R = O)* and the lower formation of ^1^O_2_ as compared to the cell-free medium. These observations reveal that most of ^3^(R = O)* is formed in the cell-free medium, while the presence of the cells in the cell suspension results in the increase of ^3^(R = O)* formation. The decrease of ^1^O_2_ formation caused by the presence of the cells in the cell suspension can be caused by two reasons. Firstly, due to the limited penetration of TMPD to the cells, the detection of ^1^O_2_ formed deep inside the cells is less likely. Secondly, the cell-free medium is more suitable environment for the reactions such as Russell mechanism know to result in the formation of ^1^O_2_. Based on this consideration, it is proposed that in the presence of cells the radicals formed in the medium oxidize the cellular components and thus less likely participate in ^1^O_2_ formation reactions.

In conclusion, the addition of H_2_O_2_ to the cell suspension results in the formation of electronically excited species by three simultaneous reactions: the oxidation of cellular components^39^,^40^,^41^,^42^, the oxidation of growth medium components, especially free amino acids and fetal bovine serum, which might further cause the oxidation of the cellular components, and the oxidation of the medium components. In this study, we mainly focused on the detection of electronically excited species and possible explanation of their formation rather than the place of their origin. The detail characterization of the site of origin of the electronically excited species will be the subject of the forthcoming study.

## Methods

### Cell Culture

**Figure 9:**
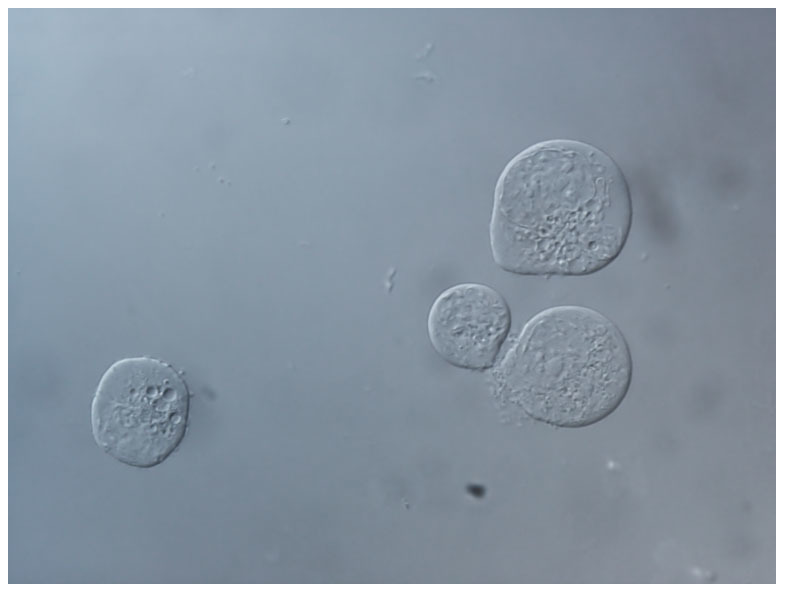
Picture of U266 cells in Nomarski DIC channel.

Human MM cell line U266 was used in this study^53^ (Fig. 9). U266 cells were grown in RPMI-1640 supplemented with 2 mM L-glutamine, 10% FBS, antibiotics at 37°C in humidified 5% CO_2_ atmosphere. Viability of the cells was measured by Trypan Blue viability test. Subsequently, 10 μl of cell suspension was mixed with 10 μl of 0.4% Trypan Blue. Cells were counted by TC20 automated cell counter (Bio-Rad Laboratories, California, USA).

### Two-dimensional ultra-weak photon emission imaging

Two-dimensional ultra-weak photon emission was detected by CCD camera installed in a temperature controlled black box placed in a black painted inner darkroom. The measurement systems inside the inner darkroom were controlled and data were recorded with the computer located in the outer darkroom. The highly sensitive CCD camera VersArray 1300B (Princeton instruments, Trenton, NJ, USA) with spectral sensitivity in the range 350 to 1000 nm and close to 90% quantum efficiency in the visible range of the spectra was used to record the 2-D spectra. Objective lens of 50 mm focal distance (F mount Nikkor 50-mm, f: 1,2, Nikon) was used to enhance the light collecting efficiency. The CCD element was cooled down to −110°C in order to reduce the dark count. The following parameters were used during the measurement: scan rate 100 kHz; gain 2; image format 1340 × 1300 pixels; binning mode 4; distance between detector and the reflecting mirror 40 cm; and accumulation time 30 min.

### One-dimensional ultra-weak photon emission

One-dimensional ultra-weak photon emission was detected by PMT system installed in a black painted inner darkroom. The measurement systems inside the inner darkroom were controlled and data were recorded with the computer located in the outer darkroom. A low-noice PMT R7518P, sensitive in the spectral range 185 to 730 nm, and a photon counting unit C9744 (Hamamatsu Photonics K.K., Iwata City, Japan) were employed to measure one-dimensional photon emission. To reduce the dark noise, PMT was cooled down to −30°C using thermoelectric cooler C9143 (Hamamatsu Photonics, K.K., Iwata City, Japan). All the measurements were recorded at −960 mV. The PMT was placed 5 cm above the sample during the measurement. In order to cut off the blue-green region of the spectra long-pass edge interference filter (600 nm) was used.

### Detection of singlet oxygen by electron paramagnetic resonance spin-trapping spectroscopy

EPR spin-trapping spectroscopy was used to monitor the formation of ^1^O_2_ in cell suspension. Hydrophilic compound TMPD was used in order to detect ^1^O_2_. To eliminate the impurity of TMPD EPR signal, the TMPD was purified twice by vacuum distillation. Cell suspension was treated with 5 mM H_2_O_2_ in the presence of 50 mM TMPD and culture medium. Cell suspension previously treated with H_2_O_2_ were put into a glass capillary tube (Blaubrand intraMARK, Brand, Germany) and EPR spectra were recorded using an EPR spectrometer MiniScope MS400 (Magnetechh GmbH, Berlin, Germany). EPR conditions were as follows: microwave power, 10 mW; modulation amplitude, 1 G; modulation frequency, 100 kHz, sweep width, 100 G, scan rate, 1.62 Gs^−1^, gain 500.

### Singlet oxygen imaging by confocal laser scanning microscopy and image analysis

SOSG (Molecular Probes Inc. Eugene, OR, USA) was applied to the U266 cells in order to visualize ^1^O_2_ production. Cell suspension treated with H_2_O_2_ was incubated with 50 μM SOSG for 30 min in dark. Following incubation, U266 cells were gently washed with 20 mM K-buffer, and consequently the ^1^O_2_ imaging was performed by a confocal laser scanning microscope, Fluorview 1000 (Olympus Czech Group, Prague, Czech Republic). The transition images were obtained by transmitted light detection module with 405 nm excitation with a near-ultraviolet (UV) laser diode and Nomarski DIC filters (Olympus). Simultaneously visualized fluorescence channel resulted from excitation by a 488 nm line of argon laser, representing the signals for SOSG fluorescence detected by 505–525 nm emission filter set. The proper intensity of lasers was set according to unstained samples at the beginning of each experiment^54^. The integral distribution of signal intensity (ranging from 0 to 4095 levels of brightness) within the images was evaluated using software FV10-ASW 3.0 Viewer (Olympus).

## Author Contributions

P.P. and M.R. designed the study; M.R. performed most of the experiments; M.S. performed the experiments including confocal laser scanning microscopy, M.R. analyzed the data, P.P. and M.R. drafted the manuscript. All authors read and approved the final manuscript.
